# Retention and mortality outcomes from a community-supported public–private HIV treatment programme in Myanmar

**DOI:** 10.7448/IAS.19.1.20926

**Published:** 2016-10-25

**Authors:** Gitau Mburu, Aung Zayar Paing, Nwe Ni Myint, Win Di, Kaung Htet Thu, Mala Ram, Christopher J Hoffmann, Bangyuan Wang, Soe Naing

**Affiliations:** 1 Programme Impact Unit, International HIV/AIDS Alliance, Brighton, United Kingdom; 2 Division of Health Research, School of Health and Medicine, Lancaster University, Lancaster, United Kingdom; 3 International HIV/AIDS Alliance in Myanmar, Yangon, Republic of the Union of Myanmar; 4 Knowledge Mobilisation and Impact Unit, Institute of Development Studies, Brighton, United Kingdom; 5 Department of Medicine, Johns Hopkins University, Baltimore, MD, USA

**Keywords:** HIV, antiretroviral therapy, private sector, public–private partnerships, Myanmar

## Abstract

**Introduction:**

There is a growing interest in the potential contribution the private sector can make towards increasing access to antiretroviral therapy (ART) in low- and middle-income settings. This article describes a public–private partnership that was developed to expand HIV care capacity in Yangon, Myanmar. The partnership was between private sector general practitioners (GPs) and a community-based non-governmental organization (International HIV/AIDS Alliance).

**Methods:**

Retrospective analysis of 2119 patient records dating from March 2009 to April 2015 was conducted. Outcomes assessed were immunological response, loss to follow-up, all-cause mortality, and alive and retained in care. Follow-up time was calculated from the date of registration to the date of death, loss to follow-up, transfer out, or if still alive and known to be in care, until April 2015. Cox proportional hazards model was used to identify predictors of loss to follow-up and mortality. Kaplan–Meier survival analysis was used to estimate survival function of being alive and retained in care.

**Results:**

The median number of patients for each of the 16 GPs was 42 (interquartile range (IQR): 25–227), and the median follow-up period was 13 months. The median patient age was 35 years (IQR: 30–41); 56.6% were men, 62 and 11.8% were in WHO Stage III and Stage IV at registration, respectively; median CD4 count at registration was 177 cells/mm^3^; and 90.7% were on ART in April 2015. The median CD4 count at registration increased from 122 cells/mm^3^ in 2009 to 194 cells/mm^3^ in 2014. Among patients on ART, CD4 counts increased from a median of 187 cells/mm^3^ at registration to 436 cells/mm^3^ at 36 months. The median time to initiation of ART among eligible patients was 29 days, with 93.8% of eligible patients being initiated on ART within 90 days. Overall, 3.3% patients were lost to follow-up, 4.2% transferred out to other health facilities, and 8.3% died during the follow-up period. Crude mortality rate was 48.6/1000 person-years; 42% (*n*=74) of deaths occurred during the pre-ART period and 39.8% (*n*=70) occurred during the first six months of ART. Of those who died during the pre-ART period, 94.5% were eligible for ART. In multivariate regression, baseline CD4 count and ART status were independent predictors of mortality, whereas ART status, younger age and patient volumes per provider were predictors of loss to follow-up. Probability of being alive and retained in care at six months was 96.8% among those on ART, 38.5% among pre-ART but eligible patients, and 20.0% among ART-ineligible patients.

**Conclusions:**

Effectively supported private sector GPs successfully administered and monitored ART in Myanmar, suggesting that community-supported private sector partnerships can contribute to expansion of HIV treatment and care capacity. To further improve patient outcomes, early testing and initiation of ART, combined with close clinical monitoring and support during the initial periods of enrolling in treatment and care, are required.

## Introduction

One of the most remarkable achievements in global health has been the rapid expansion of HIV treatment in low- and middle-income countries [1]. Currently, >15 million people are on antiretroviral therapy (ART), having risen from 1 million in 2001 [1]. In practical terms, this increase has been facilitated by a rapid expansion of facilities offering ART. In Myanmar, for example, there were 184 sites providing ART at the end of 2011 [2] compared to just 57 sites in 2008, and significant progress has been made with regard to scope and diversity of HIV services, including outreach to key populations [3].

This expansion has been aided by advancements in evidence demonstrating the benefits of early treatment [4]. In keeping with this evidence, recently updated recommendations support offering ART to everyone with HIV regardless of CD4 count [5], the immediate consequence of which is a substantial expansion in the number of ART-eligible people [1]. In Myanmar, for example, this triples the number of people eligible for ART; at the end of 2014, a total of 85,626 individuals were on treatment of the 212,000 people living with HIV [2].

Given the need to further expand ART regardless of CD4 count, the demand for clinical services is anticipated to increase. However, existing public sector and non-governmental clinics are already stretched in their ability to deliver ART services, and the development of new public sector sites to solely meet clinical HIV care needs is not feasible in many low- and middle-income countries. In such settings, providing ART services to all who need it will require a significant shift from current infrastructure of ART delivery models [6].

In Myanmar, a non-governmental organization sought to support the government to rapidly respond to a rising demand for ART services by partnering with existing private practitioners in a public–private partnership. Providing HIV care to the general public through the private sector is an approach adopted elsewhere [7] and is becoming increasingly relevant given the rising patient volume at public health services in many low- and middle-income countries [8]. Although precise figures are lacking, evidence suggests that a growing number of people in South Africa, India, Namibia, Papua New Guinea and Tanzania are already accessing ART through private providers [9–13]. In Myanmar, non-governmental organizations and the private sector contribute 50% of current ART coverage [2]. Yet there is comparatively limited literature describing outcomes of ART from private sector and public–private partnerships.

This article describes the organization and outcomes of a partnership that has been implemented over the last five years in Yangon, between the International HIV/AIDS Alliance (the Alliance) and private sector general practitioners (GPs), to provide ART. The article informs current debates regarding the extent to which the private sector should be leveraged to provide ART [14,15] and the operationalization of such an approach to complement public health facilities in ART decentralization.

## Methods

### Study setting

Yangon is the main economic centre and most populous city in Myanmar, with a population of 7.3 million inhabitants, accounting for 14% of the national population [16]. Myanmar has a concentrated HIV epidemic, with a national prevalence of 0.53% [2]. HIV is concentrated among female sex workers (6.3%), men who have sex with men (6.6%) and injecting drug users (23.1%) [2]. In this context, the Alliance has been implementing community-based HIV outreach since 2004, focussing on sex workers, men who have sex with men, and transgender people through a network of 28 community-based organizations (CBOs) in several geographical locations.

### Public–private partnership intervention

In 2009, the Alliance formed a semi-franchised partnership with 16 private sector GPs in which the GPs were contracted to provide rapid diagnostic HIV testing and clinical follow-up services to HIV-positive patients. Under this partnership arrangement, the GPs conducted HIV testing, assessed and staged HIV-positive patients according to World Health Organization (WHO) criteria, initiated ART, provided clinical HIV monitoring and management, screened for tuberculosis (TB) and monitored its treatment, diagnosed and managed sexually transmitted and other opportunistic infections, and referred patients who developed serious opportunistic infections to tertiary public government facilities for inpatient care.

In turn, the Alliance supported the GPs by way of commission-based funding equivalent to US$4 for initial consultation and registration, and an equivalent of either US$1 or 2 for each subsequent clinical follow-up without or with complications, respectively. The private providers were reimbursed for all their services by the Alliance and were not allowed to charge user fees for services offered to HIV patients. In addition, the Alliance organized continuous medical education in the form of initial and refresher HIV trainings, quarterly case reviews and HIV treatment updates, and it collected, monitored and reported ART programme data to the practitioners, funders and the Ministry of Health. The Alliance also coordinated logistical supply of ART and contracted private laboratories to provide CD4 count, viral load and other laboratory services to the GPs.

Finally, the Alliance sub-granted a network of CBOs to mobilize communities, provide HIV information to key populations, identify those at risk of HIV, provide pre-test counselling, and refer those willing to test to GPs. CBOs referred patients to the GP by using referral slips. In instances where patients came to CBOs with a HIV result in hand, they were re-tested and confirmed by GPs by using rapid diagnostic tests. To support adherence once patients were initiated on ART, CBO-based peer educators and outreach workers provided ART adherence counselling at CBO sites, conducted home visits to trace patients who had missed their appointments, and facilitated peer-led adherence meetings in communities and CBO sites.

Patients were enrolled in the programme if they lived in Yangon or its environs. In discussion with the CBO staff, patients selected a general practitioner near their residential area, where they were tested, registered and accessed long-term HIV clinical care. All people living with HIV were eligible for participation in the GP programme regardless of their health status. These programme activities as well as antiretroviral drugs were financed largely through grants from the Global Fund to Fight AIDS, Tuberculosis and Malaria (Global Fund).

### Clinical procedures

Adults were eligible for ART if they had a WHO Stage IV AIDS-defining illness irrespective of their CD4 count, or if their CD4 count was below the recommended threshold in accordance with the prevailing national guidelines. Between March 2009 and December 2012, eligibility was at a CD4 count of <200 cells/mm^3^ in keeping with the 2006 WHO recommendations [17]. In December 2012, national guidelines [18] were updated to reflect the 2010 WHO guideline recommendations [19], and eligibility was at a CD4 count of <350 cells/mm^3^. Between January 2015 and the end date of the data analyzed in this article (April 2015), eligibility was at a CD4 count of <500 cells/mm^3^.

Unless contraindicated, all adult and adolescent patients started therapy with stavudine (d4T, 30 mg bid), lamivudine (3TC, 150 mg bid) and efavirenz (EFV, 600 mg hs) or nevirapine (NVP, 200 mg bid) until December 2012, from when the following first-line regimen was used: tenofovir (TDF, 300 mg od), lamivudine (3TC, 150 mg bid) and efavirenz (EFV, 600 mg qhs). The second-line regimen was tenofovir (TDF, 300 mg od), lamivudine (3TC, 150 mg od) and ritonavir-boosted lopinavir (LPV/r, 400/100 bid) for patients on the former first-line regimen and zidovudine (AZT, 300 mg bid), lamivudine (3TC, 150 mg od) and ritonavir-boosted lopinavir (LPV/r, 400/100 bid) for patients on the latter first-line regimen.

Patients with TB and a CD4 count of >200 cells/mm^3^ were first treated for TB and then commenced on ART, whereas those with a CD4 count of <200 cells/mm^3^ were initiated on ART after completing two months of TB therapy. GPs reported patients who missed appointments or were believed to need adherence support to one of the partner CBOs for tracing or adherence support. The specific partner CBO was selected based on the residential location of the patient and CBO catchment area. Patient appointments were every two weeks for the first month, monthly from the second to the sixth month, every two months from the sixth month to one year and every three months after one year. Clients with opportunistic infections requiring acute management were referred to tertiary government facilities for inpatient care. CBOs continued to provide financial assistance and psychosocial support during hospital stay. Deaths were reported to the general practitioner by a family member or identified by a CBO outreach worker during tracing of patients who missed an appointment. Suspected deaths were confirmed by a senior staff from the CBO and were followed up with a verbal autopsy by the GP.

### Data collection

Data were routinely collected through the programme's health information system, a system that is based on Fuchia software (www.epicentre.msf.org). GPs first collected and recorded clinical data on to paper-based patient records, and these data were then transferred manually to the computerized software system on a monthly basis. To allow longitudinal monitoring of all patient outcomes, each GP was allocated a unique code that was linked to each patient under his or her care. Routinely collected baseline data included demographic data, and the results of immunological (CD4 count), haematological (full blood count), liver (alanine aminotransferase and aspartate aminotransferase) and renal (creatinine, urea) function tests. Subsequently, immunological (CD4 count) and haematological tests were performed approximately every six months as part of routine follow-up. Viral load tests were only obtained for clinically and immunologically failing patients and not for routine monitoring due to cost considerations.

### Operational definitions

The primary outcomes were loss to follow-up, CD4 count response (as a proxy for ART success given the lack of routine viral load measurements) and mortality during follow-up. The follow-up period was defined and derived from the date of registration through the pre-ART and subsequent ART periods. Date of registration was the date when a patient was enrolled in care, being the day when a patient was first seen by a GP for the initial consultation after being diagnosed positive. Patients were first identified as being at risk by CBOs, provided with referral for HIV testing, tested for HIV by GPs, and then registered for care if their results were positive. Patients whose test results were HIV negative received on-going counselling and other prevention services from CBOs.

Loss to follow-up was defined as patients who could not be traced for at least four weeks after a missed appointment with a GP, despite attempts by the CBO's outreach team to contact them. The date when a patient was classified as being lost was four weeks after the date of a missed appointment. Provider patient volume was defined as the number of HIV patients followed up by a provider during the study period. Patients only saw the provider with whom they were registered. Transfer-outs were defined as patients who had documented (either in clinic records or in CBO tracing) transfer to another facility for continuation of pre-ART or ART care. Survival time of being alive and retained in care was defined as the interval between date of registration with the GP and end of the observation period, date of loss to follow-up, death or transfer out. Baseline CD4 count was defined as the initial CD4 count at registration. The variables included in the logistic model were age, sex, WHO stage, baseline CD4 count, ART status, patient volume of provider and TB diagnosis.

### Data analysis

Data were assessed for completeness, missing values and outliers. Missing variables were obtained from primary GPs’ paper records to ensure that all patients who were registered with the GPs were included in the analysis. The final dataset was transferred to IBM SPSS statistics version 21.0. Descriptive statistics were used to summarize demographic and clinical characteristics of study patients. Association between predictor variables and loss to follow-up and mortality outcomes were explored through logistic regression, and odd ratios and their 95% confidence intervals (CIs) were derived. To facilitate this, continuous variables such as age, CD4 count, provider patient volume and baseline CD4 counts, months of follow-up and months on ART were transformed into categorical variables.

Cox proportional hazards model was used to examine factors independently associated with mortality, and results were expressed in hazard ratios (HRs) and 95% CIs, with a statistical significance assumed at *p*<0.05. Factors were included in multivariate models if they were significant at *p <*0.2 in bivariate analysis. Finally, with regard to Kaplan–Meier analysis of alive and retained outcome, survival functions at various time points between registration to death or loss to follow-up events were estimated, and Kaplan–Meier curves were illustrated. Kaplan–Meier analysis of alive and retained outcome used an intention-to-treat analysis, where death or loss to follow-up were treated as events and those transferred out were censored. Hence, the analysis adopted a worst-case scenario whereby patients who were lost to follow-up were presumed dead, consistent with reports of high rates of death among patients lost to follow-up after ART [20]. Patients who were transferred out were not assumed to have died because they had been linked to care of specific and known providers and facilities. All patients were also censored at the end of observation period.

### Ethical considerations

This study involved secondary analysis of existing medical records. De-identified patient data were abstracted into a study database, and unique study identifiers were generated for each participant. All records were held securely in a password-protected computerized database. The conduct of this study did not expose patients to unnecessary risk. The study protocol was reviewed and approved by the Ethics Review Committee of the Department of Medical Research, Ministry of Health, Republic of the Union of Myanmar (ref: ERC 00616/DMR/2016/019).

## Results

### Patient characteristics

The dataset included longitudinal records from 2119 HIV patients who were followed from the point of their respective registration to April 2015. These records were from all the patients who registered with the GPs since programme inception to the end of April 2015. Depending on their baseline CD4 counts at registration, patients spent varying periods on pre-ART and ART care. The first patients were started on pre-ART care in March 2009. The median follow-up period was 13 months [interquartile range (IQR): 7–29 months]. Of the 2119 patients included in the analysis, 90.7% (*n*=1922) initiated ART, and the remaining 9.3% (*n*=197) had only accessed pre-ART care. Of the 197 patients who were receiving pre-ART care, 165 (83.8%) were eligible for ART initiation. The median duration that patients had been on ART was 14 months (IQR: 8–30 months). Just over half (56.6%) were male, and their median age was 35 years (IQR: 30–41 years).

A small proportion of patients (5.0%) were young people aged 16 to 24 years, and a similarly small proportion (3.3%) of patients were aged over 55 years. The median CD4 count at registration was 177 cells/mm^3^, with an IQR of 162 to 304 cells/mm^3^. This median CD4 count at registration increased from 122 cells/mm^3^ in 2009 to 125, 161, 163, 198 and 194 cells/mm^3^ in 2010, 2011, 2012, 2013 and 2014, respectively. Overall, 14.4% of patients were in WHO Stage I, 11.6% were in Stage II, 62.2% were in Stage III and 11.8% were in Stage IV at registration. The median number of patients per general practitioner was 42 (IQR: 25–227) (Table 1).

**Table 1:** Characteristics of patients in the programme

Characteristic	Category	Summary measure	Frequency	Proportion
Gender	Female		920	43.4
	Male		1199	56.6
Age	Range	16–73		
	Median	35 (IQR: 30–41)		
	≤24		106	5.0
	25–39		1322	62.4
	40–54		622	29.4
	≥55		69	3.3
Baseline CD4 count at registration	Median	177 (IQR: I62–304)		
	<50		448	21.1
	50–199		696	32.8
	200–349		647	30.5
	350–499		265	12.5
	>500		63	3.0
Baseline WHO stage at registration	I		305	14.4
	II		245	11.6
	III		1318	62.2
	IV		251	11.8
TB diagnosis	Yes		691	32.6
	No		1428	67.4
Patients per GP	Median	42 (IQR: 25–227)		
	<50		232	10.9
	50–250		418	19.7
	>250		1469	69.3
Months of follow-up	Median	13 (IQR: 7–29)		
	≤6		453	21.4
	7–12		556	26.2
	13–24		443	20.9
	25–36		239	11.3
	37–48		247	11.7
	49–60		80	3.8
	61–72		101	4.8
ART status	Pre-ART (total)		197	9.3
	Ineligible		32	1.5
	Eligible		165	7.8
	On ART (total)		1922	90.7
Months on ART	Median	14 (IQR: 8–30)		
	≤6		357	18.6
	7–12		525	27.3
	13–24		433	22.5
	25–36		216	11.2
	37–48		234	12.2
	49–60		75	3.9
	61–72		82	4.3
Status	Alive in programme		1783	84.1
	Lost to follow-up		70	3.3
	Transferred out		90	4.2
	Died		176	8.3

ART, antiretroviral therapy; CD4, cluster of differentiation 4; GP, general practitioner; IQR, interquartile range; TB, tuberculosis; WHO, World Health Organization.

Provider patient volumes and outcomes across the 16 GPs are summarized in Table 2.

**Table 2:** Provider patient volumes and outcomes across general practitioners

General practitioner ID	Number of patients	Alive	Lost to follow-up	Transferred out	Total deaths	% dead
1	411	338	14	20	39	9.5
2	366	306	9	12	39	10.7
3	347	307	3	5	32	9.2
4	345	276	28	14	27	7.8
5	187	166	2	3	16	8.6
6	134	123	0	6	5	3.7
7	97	80	0	9	8	8.2
8	47	34	4	5	4	8.5
9	36	32	2	2	0	0.0
10	36	19	1	12	4	11.1
11	34	30	4	0	0	0.0
12	26	22	2	0	2	7.7
13	23	22	0	1	0	0.0
14	17	15	1	1	0	0.0
15	7	7	0	0	0	0.0
16	6	6	0	0	0	0.0

### Immunological response

**Figure 1:**
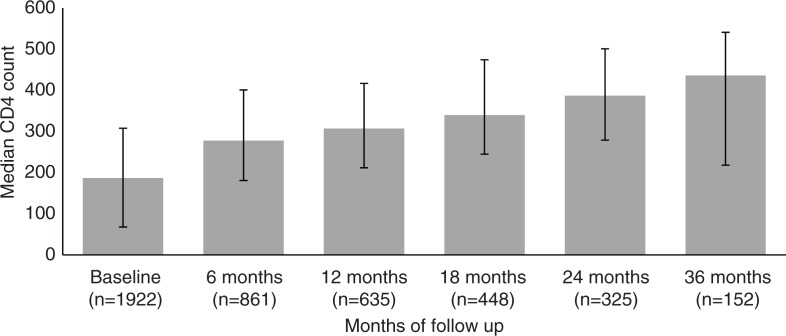
Immunological response among patients on ART.

CD4 counts of patients on ART increased by time on ART reflecting immunological improvement presumably from virologic control. The median CD4 count among those on ART increased from 187 cells/mm^3^ at baseline (*n*=1922) to 278 cells/mm^3^ at six months (*n*=861), 307 cells/mm^3^ at 12 months (*n*=635), 340 cells/mm^3^ at 18 months (*n*=448), 387 cells/mm^3^ at 24 months (*n*=325), 436 cells/mm^3^ at 36 months (*n*=152), 439 cells/mm^3^ at 48 months (*n*=75) and 482 cells/mm^3^ at 60+ months of follow-up (*n*=10) (Figure 1).

### Time to ART initiation

**Figure 2:**
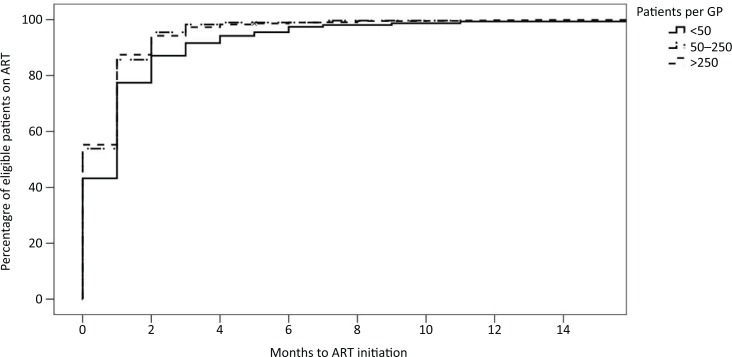
Time to ART initiation.

Of the 1642 patients who were eligible for ART at registration, 90.0% initiated ART. On average it took patients 29 days from registration to be prescribed ART for eligible patients. Of those eligible, 48.4% were initiated on ART within the first 30 days of registration, 93.8% within 90 days and 98.4% by 180 days. Results from a univariate Cox proportional hazard model suggested that compared with providers with <50 patients, providers with 50 to 250 and >250 patients initiated ART more rapidly: HR=1.243 (95% CI: 1.243–1.513; *p*=0.031) and HR=1.247 (95% CI: 1.052–1.478; *p*=0.011), respectively (Figure 2).

### Loss to follow-up

**Figure 3:**
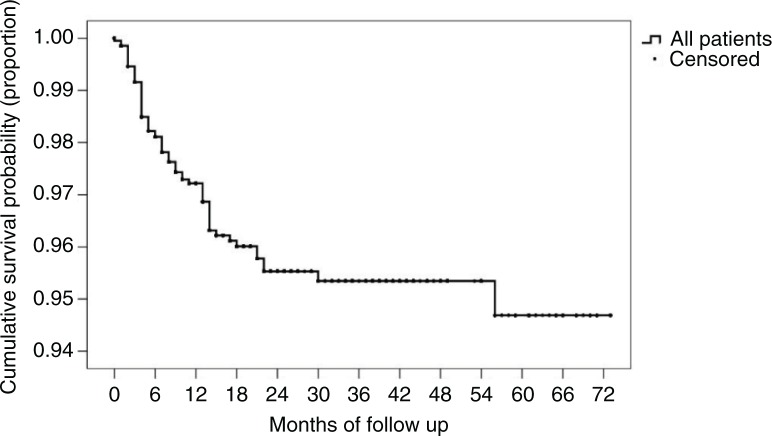
Kaplan–Meier curve of loss to follow-up.

In total, 70 (3.3%) patients were lost to follow-up. The highest rate of loss to follow-up occurred in the first 6 to 12 months, after which the rate of loss to follow-up flattened gradually (Figure 3).

### Factors associated with loss to follow-up

On bivariate analysis, ART status, age and patient volume per provider showed a trend towards being predictive of loss to follow-up, and these factors remained predictive on multivariable analysis. Compared to patients aged >55 years, young people aged up to 24 years were 13.6 times more likely to be lost to follow-up (95% CI: 1.48–62.68; *p*=0.021). Compared to patients on ART, patients who were ineligible for ART were 33.8 times (95% CI: 14.20**–**80.46; *p*≤0.001) more likely to get lost to follow-up, and those who were eligible but not yet on ART were 13.5 times (95% CI: 7.71**–**23.68; *p*≤0.001) more likely to get lost to follow-up. Data related to provider group by volume suggested that providers with very low or very high patient volumes were more likely to have patients who were lost to follow-up. However, the CIs were quite wide. Gender, CD4 at baseline, TB diagnosis and WHO stage were not associated with loss to follow-up (Table 3).

**Table 3:** Bivariate and multivariate analysis of loss to follow-up

		Bivariate analysis			Multivariate analysis		
			
Variable	Category	OR	Confidence interval	*p*	aOR	Confidence interval	*p*
Gender	Male	1.15	0.71–1.88	0.558	–	–	–
	Female	1					
Age				0.050			0.011
	≤24	5.61	0.68–45.41	0.110	13.58	1.48–62.68	0.021
	25–39	2.45	0.33–18.04	0.379	4.59	0.58–36.71	0.150
	40–54	1.68	0.22–12.92	0.618	3.00	0.36–25.05	0.310
	≥55	1			1		
Provider group				0.002			0.002
	<50 patients	13.35	3.00–59.30	0.001	15.83	3.44–72.82	<0.001
	50–250 patients	1			1		
	>250 patients	7.93	1.92–32.69	0.004	8.70	2.70–36.57	0.003
WHO stage				0.549	–	–	–
	I	1.81	0.68–4.85	0.233			
	II	1.02	0.32–3.22	0.966			
	III	1.44	0.60–3.42	0.404			
	IV	1					
ART status				<0.001			<0.001
	Pre-ART ineligible	30.93	13.77–69.46	<0.001	33.80	14.20–80.46	<0.001
	Pre-ART eligible	11.55	6.73–19.84	<0.001	13.51	7.71–23.68	<0.001
	On ART	1			1		
TB diagnosis	No	1.05	0.63–1.76	0.830	–	–	–
	Yes	1					
CD4 at baseline				0.200	–	–	–
	≤50	1					
	51–200	1.51	0.68–3.34	0.301			
	201–350	2.37	1.11–5.04	0.025			
	351–500	1.51	0.57–3.98	0.396			
	>500	1.59	0.33–7.57	0.554			

aOR, adjusted odds ratio; ART, antiretroviral therapy; CD4, cluster of differentiation 4; OR, odds ratio; TB, tuberculosis.

### Mortality

**Figure 4:**
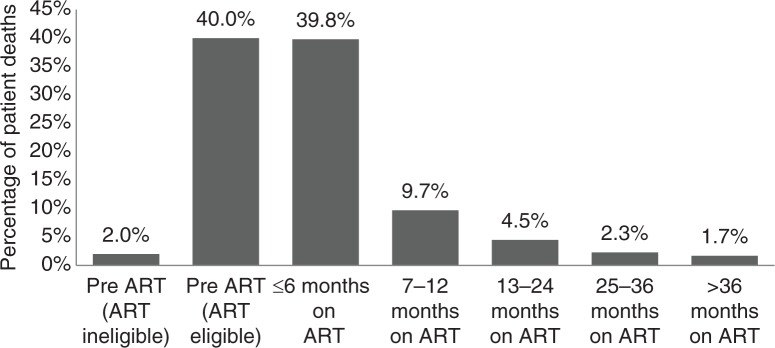
Mortality during pre-ART and ART periods.

In total, 176 (8.3%) patients died between March 2009 and April 2015. The total time of patients in the programme was 3624.83 person-years, giving an overall mortality rate of 48.6/1000 person-years. Incidence of deaths reduced progressively with time. Notably, 42% (*n*=74) of deaths occurred during the pre-ART period. Of those who died during the pre-ART period, 94.5% were eligible for ART. Of these, 97.1% (*n*=68) died within the first six months. Among those on ART, most deaths occurred soon after initiation of ART, with 39.8% (*n*=70) occurring in the first six months of ART. In addition, 9.7% (*n*=17), 4.5% (*n*=8), 2.3% (*n*=4) and 1.7% (*n*=3) of total deaths occurred between 7–12, 13–24 and 25–36+ months after initiation of ART, respectively (Figure 4).

### Factors associated with mortality

Bivariate analysis suggested there was a trend of association between mortality and gender, age, provider group, WHO stage, ART status, TB diagnosis and baseline CD4 count. However, results from multivariate analysis suggested that only ART status and baseline CD4 count were independently predictive of mortality. Compared to those with CD4 count >350 cells/mm^3^, patients with CD4 counts of <250 cells/mm^3^ were 8.6 (95% CI: 2.63–28.61) times more likely to die (Table 4).

**Table 4:** Bivariate and multivariate analysis of factors associated with mortality

		Bivariate analysis			Multivariate analysis		
			
Variable	Category	HR	Confidence interval	*p*	aHR	Confidence interval	*p*
Gender	Male	1.37	1.00–1.86	0.044	1.26	0.85–1.88	0.244
Age				0.002			0.048
	≤24	1					
	25–39	1.19	0.52–2.72	0.674	0.94	0.35–2.6	0.912
	40–54	1.75	0.75–4.04	0.189	1.32	0.47–3.70	0.592
	≥55	3.32	1.24–8.86	0.016	2.89	0.81–10.42	0.103
Provider group				0.031			0.297
	<50 patients	1			1		
	50–250 patients	1.69	0.82–3.50	0.153	1.00	0.43–2.36	0.984
	>250 patients	2.22	1.16–4.24	0.016	1.44	0.67–2.98	0.322
WHO stage				<0.001			0.203
	I	1					
	II	1.28	0.53–3.07	0.579	0.74	0.26–2.08	0.568
	III	2.78	1.46–5.31	0.002	1.29	0.60–2.77	0.511
	IV	3.89	1.92–7.89	<0.001	1.87	0.78–4.51	0.158
ART status				<0.001			<0.001
	Pre-ART: eligible	42.52	30.48–59.32		56.16	11.28–279.62	<0.001
	Pre-ART: ineligible	13.46	4.95–36.61		57.93	31.60–106.21	<0.001
	On ART	1					
TB diagnosis	Yes	1.39	1.03–1.88	0.029	0.99	0.66–1.50	0.966
	No	1					
CD4 at baseline				<0.001			<0.001
	<250	11.141	4.10–30.30		8.67	2.63–28.61	<0.001
	250–350	2.815	0.92–8.64		2.87	0.82–10.11	0.099
	>350	1			1		

aHR, adjusted hazard ratio; ART, antiretroviral therapy; CD4, cluster of differentiation 4; HR, hazard ratio; TB, tuberculosis.

### Alive and retained

Overall, 84.1% of patients ever in the programme were still alive at the end of the analysis period and 4.2% had transferred out. Excluding patients who transferred out, data suggested that gender, provider patient volume, WHO stage, ART status, TB diagnosis and baseline CD4 count were associated with being alive and retained on bivariate analysis. In multivariate analysis, provider patient volume, ART status and baseline CD4 remained independently predictive of being alive and retained in care. Compared to those ineligible for ART, pre-ART patients eligible for ART and those on ART were 1.4 (95% CI: 0.36–5.46; *p*=0.629) and 99.4 (95% CI: 28.65–344.86; *p*≤0.001) times more likely to be to be alive and retained, although the former was not statistically significant. Odds of being alive and retained generally increased with baseline CD4 count. Compared with patients with a CD4 count of <50, patients with a CD4 count >500 were 4.13 (95% CI: 0.95–18.02; *p*=0.054) times more likely to be alive and retained (Table 5).

**Table 5:** Bivariate and multivariate analysis of alive and retained

		Bivariate analysis			Multivariate analysis		
			
Variable	Category	OR	Confidence interval	*p*	aOR	Confidence interval	*p*
Gender	Female	1.32	1.00–1.74	0.047	1.111	0.78–1.57	0.551
	Male	1			1		
Age				0.099			0.114
	≤24	1			1		
	25–39	1.26	0.70–2.27	0.438	1.90	0.95–3.81	0.071
	40–54	1.03	0.56–1.90	0.911	1.58	0.76–3.27	0.217
	≥55	0.60	0.26–1.38	0.236	0.90	0.32–2.57	0.848
Provider patient volume				0.009			0.015
	<50 patients	1			1		
	50–250 patients	1.52	0.87–2.67	0.139	1.79	0.88–3.66	0.110
	>250 patients	0.82	0.52–1.29	0.402	0.83	0.48–1.45	0.513
WHO stage				0.001			0.553
	I	2.40	1.39–4.14	0.002	1.15		
	II	2.74	1.48–5.04	0.001	1.80	0.55–2.39	0.711
	III	1.32	0.90–1.92	0.145	1.20	0.80–4.07	0.158
	IV	1			1	0.74–1.93	0.465
ART status				<0.001			<0.001
	Pre-ART: ineligible	1			1		
	Pre-ART: eligible	0.49	0.15–1.55	0.227	1.40	0.36–5.46	0.629
	On ART	39.44	14.12–110.19	<0.001	99.40	28.65–344.86	<0.001
TB diagnosis	No	1.32	1.00–1.74	0.043	1.06	0.73–1.55	0.758
	Yes	1			1		
CD4 at baseline				<0.001			0.001
	≤50	1			1		
	51–200	1.76	1.27–2.44	0.001	1.43	0.95–2.16	0.083
	201–350	2.66	1.85–3.82	<0.001	2.13	1.31–3.49	0.002
	351–500	5.46	2.92–10.21	<0.001	5.77	2.27–14.66	<0.001
	>500	8.10	1.94–33.78	0.004	4.13	0.95–18.02	0.054

aOR, adjusted odds ratio; ART, antiretroviral therapy; CD4, cluster of differentiation 4; OR, odds ratio; TB, tuberculosis.

**Figure 5:**
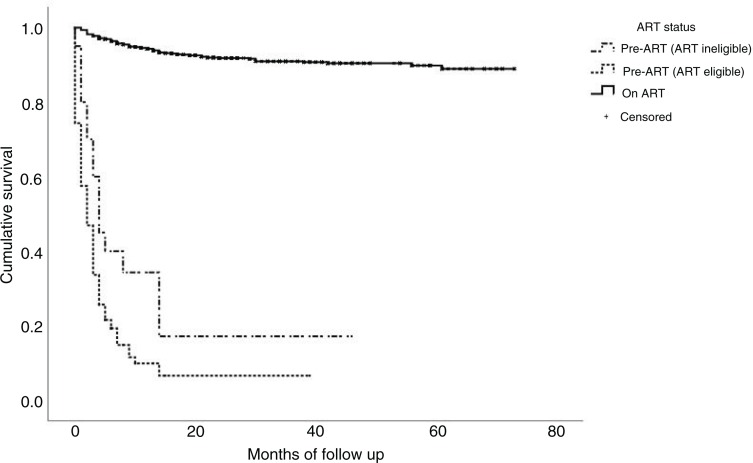
Kaplan–Meier survival function curve of being alive and retained.

An estimation of survival functions at various time points between time of registration on the programme to death or loss to follow-up event indicated that patients’ probability of survival at six months after registration was 91.9% among all patients, 96.8% among patients on ART, 38.5% among ART-ineligible patients and 20.0% among pre-ART but ART-eligible patients. At 12 months, the probability of survival overall was 89.0% among all patients, 94.2% among patients on ART, 32.5% among ART-ineligible patients and 9.7% among pre-ART but ART-eligible patients (Figure 5).

## Discussion

This study demonstrates the outcomes of ART administered and monitored by GPs in the urban setting of Yangon in Myanmar. ART was provided within a partnership between private sector GPs and a local non-governmental organization. In this model, 3.3% of 2119 patients were lost to follow-up, 4.2% transferred out to other health facilities and 8.3% died during the follow-up period of March 2009 to April 2015. The findings of high pre-ART and early ART mortality reinforce prior reports on the substantial risk of mortality among these populations. Our finding is that among a diverse group of GPs managing different volumes of people living with HIV, outcomes were overall good.

A critical measure of clinic performance regarding HIV care is time to ART initiation. Among the GPs in this study, it was a mean of 29 days. This is a substantially shorter time to ART than reports from other low- and middle-income settings. For example, despite guidelines specifying rapid ART initiation among people presenting with a CD4 count <200 cells/mm^3^ in South Africa time to ART initiation is slow and 50 to 72% of ART-eligible patients are initiated on ART within 6 to 12 months [21,22].

In addition, once diagnosed and registered in care, rates of loss to follow-up were low compared to other settings. Studies among private GPs in urban and rural South Africa found relatively high rates of loss to follow-up, ranging from 15% at 12 or 24 months [23,24] to 50% at 36 months [25]. In Myanmar, other providers of ART have also reported higher loss to follow-up rates of rates ranging from 6.5 to 7% [26,27]. The strong community component that facilitated active tracing of those who missed appointments and provision of psychosocial support to all patients through CBOs may have contributed to low loss to follow-up, despite a strict definition of loss to follow-up being used in the programme whereby a cutoff of four weeks was used, a time that is shorter than the commonly used 60 days [28] and the recommended 180 days [29]. Consistent with other studies, ART status [27], younger age [26,30,31], and patient volumes per provider [27,32] were associated with loss to follow-up.

The mortality rate of 8.3% found in our sample was lower than that reported from private providers in South Africa, of 18% at 12 months [23], and slightly lower than those previously reported from other large cohorts in Myanmar, which ranged from 9 to 13.8% [26,27]. This mortality rate could be an underestimate given recent findings from two reviews suggesting that more than one-third of those lost to follow-up in sub-Saharan Africa die soon afterward [33,34]. However, the generalizability of the proportion of lost to follow-up patients that die in Myanmar is uncertain, given that most data in the above-mentioned reviews were from sub-Saharan Africa. Therefore, an adjusted mortality rate assuming an excess mortality among our cohort could not be determined. Nevertheless, given the low absolute number of loss to follow-up, mortality would still be low even if this assumption of one-third were made. Estimation of crude mortality rate taking into account person-years at risk might provide better estimates of mortality rate. In our study, this was 48.6/1000 person-years. In the survival analysis of alive and retained outcomes, we used an intention-to-treat approach whereby all lost to follow-up patients were presumed dead, and although, this approach may underestimate survival functions, our findings demonstrated high survival rates. At 12 months, the probability of being alive and retained was 89.0% among all patients.

Although the overall mortality was low, mortality was high during pre-ART and the first six months of ART, similar to observations in other studies [35,36]. Probability to be alive and retained was especially low among both ART-eligible pre-ART patients and ART-ineligible patients (9.7 and 32.5% at 12 months, respectively). This suggests that efforts to prevent early deaths should particularly be focussed on ART-eligible pre-ART patients, whereas additional emphasis to prevent loss to follow should be focussed on ART-ineligible patients.

Early initiation of ART regardless of CD4 count will reduce mortality given the protective impact of ART on mortality [4]. Once initiated on ART, close clinical monitoring and support during the initial 6 to 12 months after enrolment in ART will be required. A recent study from Kenya showed that frequent monitoring through weekly or biweekly rapid contacts with high-risk patients during the early months of ART can significantly reduce mortality [37]. Similar to other studies [24,36], ART and higher baseline CD4 count reduced risk of death and getting lost to follow-up, re-emphasizing the need for early testing and ART initiation.

An important aspect of this study was related to the impact of patient volume per GP on patient outcomes. Although some differences in patient outcomes became non-significant when controlled for other factors in multivariate analysis (such as mortality), our data suggested that provider volume could have an independent impact on survival and loss to follow-up. In addition, although 93.8% of patients were being initiated on ART within 90 days of becoming eligible, lower volume providers were slower in initiating ART and may need additional support and training to become comfortable with prompt ART initiation. These findings add to existing literature about the importance of physician characteristics on HIV care outcomes [32,38], and they suggest the need for volume-based tailored support to providers to optimize treatment outcomes. However, consideration should be given to the heavy engagement of CBO in influencing some patient outcomes (such as loss to follow-up) before firm conclusions are made.

### Implications

Although there is an ongoing and perhaps never-ending ideological debate regarding whether the private sector should be leveraged or invested in to provide ART [14,15], findings from this study have important implications for practice and policy. First, given the current commitment to expand ART decentralization in Myanmar [3], private sector GPs could contribute to achieving this goal.

Second, there are valid questions regarding sustainability of this model. One of the concerns regarding access to treatment in the private sector is out-of-pocket regimen costs [39] that can interfere with continuity of care [9], frequency of clinical monitoring visits [15], regimen choice [40], ART adherence [40] and, ultimately, viral suppression [41,42]. In our partnership model, there were no out-of-pocket payments made by patients as all their diagnostic and clinical care costs were covered through the partnership. The protocols for clinical appointments and laboratory tests were pre-approved by the technical staffs from the partner non-governmental organization, which prevented unnecessary tests and minimized costs. Nevertheless, this partnership and most other HIV treatment programmes in Myanmar are financed through external funding, mainly the Global Fund. Experiences from South Africa suggest that direct government support and employment-supported insurance schemes could cover the cost of antiretroviral services [43] and that this and other options for enhancing social protection could be explored for genuine sustainability in Myanmar.

Third, evidence suggests that formal acknowledgement, interaction, engagement and regulation of the private sector improves quality of care [44]. Although ministries of health are best suited to perform these functions, it is more difficult to establish close-knit quality-assurance initiatives among private practices compared to the public sector [45,46]. The partnership described in this article allowed a system of quality assurance and skills-based training to be implemented with a loosely franchised group of private practices. Other research has suggested that quality assurance linked with franchising and accreditation of provider networks could be an important aspect of private sector GP outcomes [43]. This is relevant given that private practitioners who are already providing HIV care frequently lack knowledge of current concepts of HIV management [47] and are often in need of training and skills-based capacity building [45,47,48]. More broadly, and building on this partnership, system-wide strengthening efforts implemented by the government that link and address both the private and public sectors could enable leveraging investments in HIV to build sustainable health systems for managing large cohorts of patients receiving HIV-related care. These efforts could focus on areas of documented concerns related to private sector practitioners, including adherence to guidelines [15,49], integration of other conditions such as TB and mental health with chronic HIV care [46,50], and prescription and polypharmacy patterns [50,51].

## Conclusions

In the context of rising numbers of people in need of ART within a context of overloaded public health systems, this study documents the outcomes of HIV treatment provided within a partnership between private sector GPs and a local non-governmental organization that incorporated community-based psychosocial support. In this study, viral load was not routinely available due to associated costs, which is a limitation of the extent to which conclusions can be drawn regarding the quality of care outcomes, especially in light of recent recommendations for routine viral load monitoring [5]. However, studies conducted among private sector practitioners where viral load testing is routinely available have reported viral suppression rates of up to 82% at 36 weeks among retained patients [25]. Although generalizability of the findings in this study may be limited by context specific factors such as heavy involvement of community-based support, results from this study contribute to the literature describing outcomes of ART from the private GPs and indicate potential for wider engagement of the private sector and community support towards achieving universal access to decentralized ART.
